# Issue Information

**DOI:** 10.1002/cre2.119

**Published:** 2019-10-30

## Abstract

No abstract is available for this article.

